# Vascular Sap Proteomics: Providing Insight into Long-Distance Signaling during Stress

**DOI:** 10.3389/fpls.2016.00651

**Published:** 2016-05-12

**Authors:** Philip Carella, Daniel C. Wilson, Christine J. Kempthorne, Robin K. Cameron

**Affiliations:** Department of Biology, McMaster UniversityHamilton, ON, Canada

**Keywords:** abiotic stress, biotic stress, long-distance signaling, phloem, proteomics, xylem

## Abstract

The plant vascular system, composed of the xylem and phloem, is important for the transport of water, mineral nutrients, and photosynthate throughout the plant body. The vasculature is also the primary means by which developmental and stress signals move from one organ to another. Due to practical and technological limitations, proteomics analysis of xylem and phloem sap has been understudied in comparison to accessible sample types such as leaves and roots. However, recent advances in sample collection techniques and mass spectrometry technology are making it possible to comprehensively analyze vascular sap proteomes. In this mini-review, we discuss the emerging field of vascular sap proteomics, with a focus on recent comparative studies to identify vascular proteins that may play roles in long-distance signaling and other processes during stress responses in plants.

## Introduction

Plants are unable to relocate during unfavorable environmental conditions and instead rely on intricate signaling pathways that work at the local and systemic level to withstand stress. At the local level, stress-related signals move cell-to-cell through plasmodesmata (symplastic) or diffuse through the extracellular space (apoplastic). These communication processes are effective for signaling between neighboring cells or adjacent tissues, but are insufficient for systemic communication between organs (Lucas et al., 2013). For long-distance movement between distant tissues, macromolecules often access the plant vasculature; a system of specialized transport conduits connecting all organs of the plant.

### The Plant Vascular System: Sharing Resources and Information

The xylem vessels and phloem sieve tubes of the plant vascular system connect above- and below-ground organs, allowing vascular plants to draw from two distinct resource pools. The xylem provides an avenue for the unidirectional transport of water and mineral nutrients (xylem sap) from roots to aerial tissues that is driven by the transpiration stream. In contrast, the phloem allows for the bidirectional movement of photosynthate and other macromolecules throughout the plant, from areas of synthesis or excess (source) to areas of use (sink) such as storage tissues or zones of active growth. The fluid within the phloem is known as phloem sap, and its movement is thought to be driven by a hydrostatic pressure gradient along sieve tubes according to the pressure-flow hypothesis (reviewed in De Schepper et al., 2013; Lucas et al., 2013). However, this idea is not fully supported as data demonstrating differential pressure between source and sink phloem has not been obtained (discussed in Knoblauch and Oparka, 2012).

In addition to its role in resource allocation, the vasculature serves as an important conduit for the exchange of information between organs. Phloem sap appears to be highly complex, containing a diverse set of molecules such as sugars, lipids, amino acids, peptides, proteins, coding and non-coding RNAs, small molecules, mineral nutrients, and phytohormones (Lough and Lucas, 2006; Turgeon and Wolf, 2009; Lucas et al., 2013). By comparison, xylem sap appears to be less complex, primarily containing mineral nutrients, peptides, proteins, and hormones (Lucas et al., 2013; Turnbull and Lopez-Cobollo, 2013). Many of these molecules have been implicated as signals or signal chaperones in a number of developmental and stress-related long-distance signaling pathways such as photoperiod-induced flowering, systemic acquired resistance (SAR), induced systemic resistance (ISR), wound responses, RNA silencing, autoregulation during plant-rhizobia symbioses, and systemic nutrient starvation responses. Most of these responses have been reviewed elsewhere (Lough and Lucas, 2006; Champigny and Cameron, 2009; Turgeon and Wolf, 2009; Lucas et al., 2013; Turnbull and Lopez-Cobollo, 2013; Lin et al., 2014), therefore we focus only on those that best illustrate how xylem and phloem facilitate inter-organ communication. The photoperiod-induced flowering response serves as the standard example for long-distance signaling in plants. Many plants sense increasing day length as a cue for the transition from vegetative to reproductive development. Early experiments demonstrated that a signal moves in the phloem from leaves, where day length is perceived, to the shoot apical meristem (SAM) where the transition to flowering is initiated (Zeevaart, 1976). More recent experiments identified the phloem-mobile signal as FT (FLOWERING LOCUS T) protein (Corbesier et al., 2007; Jaeger and Wigge, 2007). Interestingly, FT interacts with phospholipids that are important for FT’s function in flowering-time regulation in the SAM (Nakamura et al., 2014). The lipid transfer protein DIR1 (DEFECTIVE IN INDUCED RESISTANCE 1) is an additional lipid-binding protein implicated in phloem-mediated long-distance signaling. During the induction of SAR, DIR1 and phloem-mobile immune signals move from locally infected to naïve distant leaves to protect against future pathogen infection (Champigny and Cameron, 2009; Champigny et al., 2013). Several hydrophobic small molecules (azelaic acid, glycerol-3-phosphate, dehydroabietinal, pipecolic acid) have been identified as potential SAR mobile signals in the phloem; however, further experimentation is required to understand how these molecules participate in SAR (discussed in Dempsey and Klessig, 2012). Xylem-mediated long-distance signaling has been implicated in certain abiotic stress responses. For example, nutrient deprivation induces xylem-mobile hormone signals (cytokinins and strigolactones) that travel from roots to shoots to alter plant development (Lucas et al., 2013). Long-distance signaling responses sometimes utilize both xylem and phloem for signal movement. Following infection with symbiotic rhizobacteria, plants produce long-distance signals that travel through the xylem from roots to shoots to inform autotrophic tissues of the impending symbiotic association. Upon the perception of this xylem-borne signal, a shoot-generated signal accesses the phloem and travels back to the roots to regulate the development of symbiotic structures (Staehelin et al., 2011). Xylem-borne CLE (CLAVATA3/EMBRYO SURROUNDING REGION-RELATED) peptide signals from roots are believed to interact with LRR–RLKs (leucine rich repeat–receptor like kinases) in shoots, which in turn induce the accumulation and movement of unidentified shoot-derived signal(s) back to roots (Searle et al., 2003; Staehelin et al., 2011; Wang et al., 2016). Together, these responses illustrate how the plant vasculature acts a conduit for information sharing between distant tissues.

### Collecting Vascular Sap

Our current understanding of vasculature-mediated long-distance signaling is impacted by the challenge of obtaining pure vascular sap. Standard methods involve collecting the fluid that exudes from the cut ends of petioles or puncture wounds of stems. Phloem sap is easily collected from cucurbits and legumes, which exude large amounts of phloem sap from petiole cut ends and stems (discussed in Turgeon and Wolf, 2009). Notably, the purity of cucurbit exudates has recently come into question, as some members of this family exude primarily from extrafascicular phloem (non-transport), and xylem (Zhang et al., 2010, 2012; Zimmerman et al., 2013). In other plants, phloem sap is collected over the course of several hours by submerging petiole ends in EDTA-containing solutions, which prevents sieve element occlusion by limiting the availability of Ca^2+^ (King and Zeevaart, 1974). While EDTA-facilitated exudation enables phloem sap collection from a wide variety of plants, the sample is substantially diluted during the process and prolonged exposure to EDTA may lead to intracellular (non-phloem) contamination caused by tissue softening. A recent improvement to this method was described by Guelette et al. (2012), who demonstrated that an initial 1-h incubation of *Arabidopsis* petioles in EDTA, followed by a 9-h exudation period in sterile water, was sufficient for metabolomics and proteomics analysis. Another technique for the collection of phloem sap is aphid stylectomy, which uses phloem-feeding insects as tools to collect pure sap directly from phloem cells (discussed in Turgeon and Wolf, 2009). While the collected phloem sap is much less dilute, collection volumes are low and the method is technically challenging.

Xylem sap is typically sampled from the cut stems of larger plants such as *Brassica oleracea, Zea mays*, and *Glycine max* via bleeding or root pressure techniques. Bleeding techniques sample xylem sap directly from the cut end of stems or petioles, while root pressure techniques apply pressure (mechanically or through positive pressure using ice) to the rootstock to force liquid through the xylem, which is then collected from the cut end of the stem (Alexou and Peuke, 2013). Despite measures such as the pre-washing and blotting of cut stems, contamination of xylem sap by phloem and/or other cellular contents can be an issue for most xylem sap collection methods (Alexou and Peuke, 2013). Since small proteins and peptides have been implicated in long-distance signaling in the xylem (Neumann, 2007; Lucas et al., 2013), Okamoto et al. (2015) recently optimized a gel-free purification technique to enrich for small proteins/peptides in xylem sap. Combining *o*-chlorophenol extractions and HPLC (high performance liquid chromatography) separation, the authors identified small proteins and peptides that were not detected using electrophoresis based-methods (Okamoto et al., 2015). However, a major limitation of these methods is the inability to collect xylem sap from smaller plants, which are often used as molecular-genetic model systems.

## Proteomics Analysis of Vascular Sap

Proteins play an important role in vasculature-mediated long-distance signaling responses, as demonstrated by the involvement of FT and DIR1 in photoperiod-induced flowering and SAR, respectively. Over the past 10 years, a number of proteomics studies provided information about the protein composition of xylem and phloem sap. Most studies relied on gel-based separation techniques such as 1D or 2D SDS-PAGE followed by standard protein detection approaches. This includes liquid chromatography (LC) coupled to mass spectrometers (MS), which consist of an ionization source (MALDI – *matrix-assisted laser desorption/ionization*, or ESI – *electrospray ionization*), and at least one of four types of mass analyzer; FTIC (fourier transform ion cyclotron), ion trap, TOF (time-of-flight), and quadrupole. Combinations of different mass analyzers in tandem MS set-ups improved protein coverage by overcoming particular weaknesses associated with each analyzer. In this review, we refer to these techniques simply as LC–MS/MS; for a comprehensive proteomics review, see Yates et al. (2009). Proteomics techniques have identified proteins in xylem and phloem sap collected from healthy plants growing in normal conditions (**Table 1**). In a recent review, Rodriguez-Celma et al. (2016) analyzed most of these studies and concluded that in general, the vascular fluids of multiple species contain proteins that appear to function in structural maintenance of the vasculature (e.g., cell wall metabolism) as well as constitutive defenses against pathogens (e.g., pathogenesis-related [PR] proteins, chitinases, proteases).

**Table 1:** Vascular sap proteomes of healthy plants.

Species	Sap collection method	Number of proteins identified	Reference
**Xylem sap proteomes**
*Brassica napus*	Root pressure	10	Buhtz et al., 2004
	Root pressure	69	Kehr et al., 2005
*Brassica oleracea*	Root pressure	10	Buhtz et al., 2004
	Bleed (stem)	189	Ligat et al., 2011
*Cucurbita maxima*	Root pressure	11	Buhtz et al., 2004
*Cucumis sativus*	Root pressure	9	Buhtz et al., 2004
*Glycine max*	Pressure (syringe)	24^1^	Djordjevic et al., 2007
	Pressure (syringe)	16	Krishnan et al., 2011
*Gossypium hirsutum*	Root pressure	455^2^	Zhang et al., 2015
*Oryza sativa*	Root pressure	118	Aki et al., 2008
*Vitis vinifera*	Bleed, pressure	7	Aguero et al., 2008
*Zea mays*	Root pressure	59	Alvarez et al., 2006
**Phloem sap proteomes**
*Arabidopsis thaliana*	EDTA-facilitated	377	Batailler et al., 2012
	EDTA-facilitated	65	Guelette et al., 2012
*Brassica napus*	Puncture	140	Giavalisco et al., 2006
*Cucurbita maxima*	Puncture	29	Walz et al., 2004
	Cut stem	1121	Lin et al., 2009
	Cut stem	47	Cho et al., 2010
	Cut stem/petioles	320	Frohlich et al., 2012
*Cucumis melo*	Cut stem/petioles	14^3^	Malter and Wolf, 2011
*Cucumis sativus*	Puncture	16	Walz et al., 2004
*Hordeum vulgare*	Stylectomy	7	Gaupels et al., 2008
*Lupinus albus*	Puncture	86	Rodriguez-Medina et al., 2011
*Lupinus texensis*	Puncture	98	Lattanzio et al., 2013
*Oryza sativa*	Stylectomy	107	Aki et al., 2008
*Ricinus communis*	Puncture	18	Barnes et al., 2004

*^1^Comparative study where treated and healthy plants displayed identical protein profiles, ^2^peptide fragments, ^3^identified as differentially abundant in different tissues of healthy plants*.

## Comparative Proteomics Analysis of Vascular Sap

Comparative proteomics of vascular sap is an excellent approach to identify proteins that may be involved in long-distance signaling responses. Early comparative proteomics studies relied on 1- or 2D difference gel electrophoresis (DIGE) techniques to compare the protein profiles of different samples. Spots present in some samples but not others are excised from the protein gel and analyzed by LC–MS/MS. Unfortunately, this type of analysis performs poorly with more complex samples, since individual proteins cannot be resolved by electrophoresis. Moreover, DIGE techniques have limited coverage since only differentially abundant proteins are analyzed (Gemperline et al., 2016). Modern gel-free comparative techniques overcome both of these issues while also providing quantitative information for every protein that is identified. Gel-free approaches such as ICAT (isotope-coded affinity tag), ICPL (isotope-coded protein labeling), or iTRAQ (isobaric tags for relative and absolute quantitation) rely on the addition of chemical labels to protein samples. Alternatively, label-free comparative proteomics, in which separate MS runs are aligned and compared *in silico*, is used to identify differentially abundant proteins between samples/treatments. Label-free quantitation is much less labor intensive compared to label-dependent techniques such as iTRAQ, and often allows for superior protein detection and more accurate quantitation (Patel et al., 2009; Trinh et al., 2013). To date, each of these techniques has been successfully employed for comparative proteomics analysis of plant vascular sap during stress responses (**Table 2**). More detailed descriptions of these methods can be found in other reviews (Yates et al., 2009; Lottspeich and Kellermann, 2011).

**Table 2:** Comparative proteomics studies of vascular sap collected during stress.

Species	Treatment	Comparative method	Total proteins	Increased abundance	Decreased abundance	Reference
**Xylem sap**
*Solanum lycopersicum*	*Fusarium oxysporum*	1D-DIGE^1^	N/A^2^	7	N/A	Rep et al., 2002
		2D-DIGE	33	25	0	Houterman et al., 2007
		Label-free^3^	285	102	156	Gawehns et al., 2015
*Brassica oleracea*	*Fusarium oxysporum*	Label-free	155^4^ 204^5^	34^4^ 61^5^	78^4^ 104^5^	Pu et al., 2016
*Glycine max*	*Fusarium virguliforme*	Presence/ Absence	112	6^6^ 5^7^	5	Abeysekara and Bhattacharyya, 2014
	*Bradyrhizobium japonicum*	2D-DIGE	19	4	3	Subramanian et al., 2009
	*Phytophthora sojae* elicitor			2	0	
*Brassica napus*	*Verticillium longisporum*	1D-DIGE	N/A	3	N/A	Floerl et al., 2008
*Zea mays*	Drought	2D-DIGE	39	33	8	Alvarez et al., 2008
	N-supply	2D-DIGE	23	14	9	Liao et al., 2012
*Brassica oleracea*	NaCl	2D-DIGE	40	22	18	Fernandez-Garcia et al., 2011
*Gossypium hirsutum*	K-limitation	Label-free	285^8^	5	41	Zhang et al., 2016
**Phloem sap**
Hybrid Poplar	Wounding	2D-DIGE	48	2	0	Dafoe et al., 2009
*Cucurbita maxima*	Wounding	ICPL^9^	300	25^10^ 39^11^	26^10^ 12^11^	Gaupels et al., 2012
*Cucumis sativus*	NaCl	iTRAQ^12^	745	25^13^ 12^14^	44^13^ 53^14^	Fan et al., 2015
*Brassica napus*	Fe-limitation	2D-DIGE	263^15^	19	22	Gutierrez-Carbonell et al., 2015
*Oryza sativa*	Brown Planthopper	iTRAQ	238	8^16^ 10^17^	26^16^ 13^17^	Du et al., 2015
*Cucumis melo*	Melon Necrotic Spot Virus	2D-DIGE	1046^15^	13	9	Serra-Soriano et al., 2015
*Arabidopsis thaliana*	*Pseudomonas syringae*	Label-free	564	16	46	Carella et al., 2016

*^1^Difference gel electrophoresis, ^2^not applicable, ^3^label-free quantitative proteomics, ^4^cultivar resistant to Fusarium oxysporum, ^5^cultivar susceptible to F. oxysporum, ^6^host-derived, ^7^pathogen-derived, ^8^peptide fragments, ^9^isotope-coded protein labeling, ^10^collected 3 h post wounding, ^11^collected 24 h post wounding, ^12^isobaric tags for relative and absolute quantitation, ^13^salt-intolerant cultivar, ^14^salt-tolerant cultivar, ^15^spots on protein gel, ^16^cultivar susceptible to insects, ^17^cultivar resistant to insects*.

### Comparative Proteomics Analysis of Xylem Sap

Several comparative proteomics studies have been performed on xylem sap collected from plants experiencing stress (**Table 2**). The first was performed by Rep et al. (2002), who used MALDI-TOF-MS peptide fingerprinting to identify seven PR-related proteins that accumulate in xylem sap of tomato (*Solanum lycopersicum*) plants infected with the xylem-infecting fungal pathogen *Fusarium oxysporum.* This finding was later supported by a 2D-DIGE proteomics study that identified host PR proteins as well as pathogen-derived proteins in tomato xylem sap during *F. oxysporum* infection (Houterman et al., 2007). Recently, more comprehensive analyses of xylem sap collected during host interactions with *F. oxysporum* were performed using label-free quantitative proteomics in tomato and *Brassica oleracea* (Gawehns et al., 2015; Pu et al., 2016). Both studies identified substantially more total proteins (∼150–285) with a relatively high proportion of those proteins showing differential abundance during infection (Gawehns et al., 2015; Pu et al., 2016). Other xylem sap proteomes collected during plant–microbe interactions include *Glycine max* infected with *Fusarium virguliforme* (Abeysekara and Bhattacharyya, 2014), *G. max* during symbiosis with *Bradyrhizobium japonicum* or during treatment with elicitors from the pathogen *Phytophthora sojae* (Subramanian et al., 2009), and *Brassica napus* infected with *Verticillium longisporum* (Floerl et al., 2008). A common theme among these proteomes is the accumulation of PR proteins such as chitinases and glucanases, which may serve in an antimicrobial capacity to limit the spread of infection via xylem vessels (Sels et al., 2008).

Comparative proteomics analysis of xylem sap during abiotic stress has been studied in *Zea mays, B. oleracea*, and *Gossypium hirsutum.* Alvarez et al. (2008) analyzed xylem sap collected from well-watered and drought-stressed *Z. mays* using 2D-DIGE and LC-MS/MS, identifying 33 proteins that accumulated during drought and 8 that decreased in abundance. Fernandez-Garcia et al. (2011) analyzed xylem sap proteomes of salt-stressed and control *B. oleracea* plants using 2D-DIGE comparative proteomics, identifying 22 proteins that accumulated during salt stress and 18 proteins that decreased in abundance. More recent comparative proteomics analyses of xylem sap have focused on responses to nutrient limitation. Liao et al. (2012) analyzed the *Z. mays* xylem sap proteome during nitrogen-limiting and -oversupply conditions using 2-DIGE and Zhang et al. (2016) performed label-free quantitative proteomics on xylem sap collected from cotton (*G. hirsutum*) seedlings grown under normal and potassium (K)-limited conditions. Interestingly, the differential abundance of PR proteins, proteases, redox-associated proteins, and cell wall metabolism proteins was observed in each of these studies (Alvarez et al., 2008; Fernandez-Garcia et al., 2011; Zhang et al., 2016), which hints at the importance of these proteins in the xylem during abiotic as well as biotic stress.

### Comparative Proteomics Analysis of Phloem Sap

Comparative proteomics analysis of phloem sap has been performed to investigate long-distance signaling during a number of stress responses (**Table 2**). Using iTRAQ-based proteomics Du et al. (2015) analyzed phloem sap collected from resistant and susceptible rice (*Oryza sativa*) cultivars that were either unexposed or exposed to phloem-feeding brown plant hopper (BPH) insects. They found that carbohydrate and protein metabolism proteins accumulated in phloem sap of susceptible BPH-infested plants, and that defense-related proteins accumulated in BPH-resistant plants. Responses to virus infection were investigated using phloem sap collected from melon plants (*Cucumis melo*) that were uninfected or infected with melon necrotic spot virus (MNSV). Using 2D-DIGE and LC-MS/MS, the authors identified a number of cell-death and redox-associated proteins that were differentially abundant during infection with MNSV (Serra-Soriano et al., 2015). Lastly, a label-free quantitative proteomics study was recently undertaken by our group to identify differentially abundant proteins in phloem sap of *Arabidopsis thaliana* during the induction of SAR with virulent or avirulent strains of *Pseudomonas syringae* (Carella et al., 2016). Of the 564 proteins identified in *Arabidopsis* phloem sap, 16 accumulated and 46 decreased in abundance during SAR. Proteins that accumulated in phloem sap during SAR included PR-1, redox-associated proteins, and putative lipid-binding proteins, while proteins with decreased abundance were associated with metabolism. The functional relevance of these proteins was investigated by performing SAR assays on corresponding T-DNA knockout mutants, which identified m-type thioredoxins (TRXm1 and TRXm4) and a major latex protein (MLP) as novel phloem-localized proteins that play functional roles in SAR.

Comparative proteomics analyses of phloem sap collected during abiotic stress responses have also been performed. An initial comparative study of phloem sap collected from hybrid poplar (healthy or mechanically wounded) identified 48 total proteins using 2D-DIGE and LC-MS/MS, with two proteins accumulating during wounding stress (Dafoe et al., 2009). A subsequent ICPL-based study of phloem sap collected from wounded and unwounded cucumber (*Cucumis sativus*) identified substantially more total and differentially abundant proteins (Gaupels et al., 2012). Interestingly, PR-type proteins were identified in the phloem sap of poplar and cucumber (Dafoe et al., 2009; Gaupels et al., 2012) and cucumber phloem also contained cyclophilins, carbon metabolism-related proteins, and other defense-related proteins (Gaupels et al., 2012). The phloem sap proteomes of plants experiencing other types of abiotic stress have been investigated in cucumber and *B. napus*. An iTRAQ-based study of phloem sap collected from salt-stressed cucumber identified several salt-responsive phloem proteins in salt-tolerant and -intolerant cultivars (Fan et al., 2015). In addition, comparative 2D-DIGE and LC–MS/MS analysis of phloem sap collected from Fe-deficient and control *B. napus* plants identified a number of redox-associated proteins with differential abundance during Fe stress (Gutierrez-Carbonell et al., 2015).

### Common Proteins Present in Vascular Sap Proteomes during Stress

Comparative proteomics analysis of vascular sap has revealed a great deal about how the vascular system responds to stress. Surprisingly, a common theme among many xylem and phloem sap proteomes collected from stressed plants is the accumulation of PR proteins including thaumatin-like proteins, chitinases, glucanases, and MLPs. This may indicate that PR proteins play a role in the protection of the vasculature against pathogen/herbivore attack. Redox-related proteins (thioredoxins, peroxidases, etc.) are similarly associated with stress responses in the vasculature. It has been proposed that these proteins play a protective role in the phloem by preventing damage to proteins caused by oxidative stress (Walz et al., 2002). This may be especially important in phloem sieve elements as these cells lack protein synthesis machinery and cannot quickly replace proteins damaged during stress. In addition, cyclophilins, glycine-rich proteins (GRPs), and putative lipid-binding proteins are commonly identified in both xylem and phloem sap proteomes. The identification of multiple lipid-binding proteins in vascular sap supports the idea of lipid-based long-distance signaling in the vasculature (Benning et al., 2012; Barbaglia et al., 2016). The role of cyclophilins and GRPs in the vasculature is less clear. Extracellular GRPs have been linked to cell wall formation, while intracellular GRPs and cyclophilins have been implicated in a number of functions including RNA-binding/chaperoning, which may suggest a role in RNA-mediated long-distance signaling in the vasculature (Mangeon et al., 2010; Kumari et al., 2013; Rodriguez-Celma et al., 2016).

## Conclusion and Future Directions

The emergence of sophisticated proteomics techniques has led to a new era in vascular sap proteomics studies. Together, these studies provide fundamental insights into the nature of plant vascular sap under normal and stress conditions. Although significant progress has been made in this field, much remains to be discovered. Further improvements to existing proteomics techniques will make it possible to detect low-abundance proteins essential for vasculature function. Moreover, novel xylem sap collection protocols for genetically tractable model systems are needed to investigate the importance of xylem-mobile proteins at the molecular-genetic level. In the meantime, researchers can take advantage of the *Arabidopsis* model system and the recently improved *Arabidopsis* phloem sap collection method (Guelette et al., 2012; Tetyuk et al., 2013) to expand our understanding of the molecular mechanisms of phloem-mediated long-distance signaling during plant stress.

## Author Contributions

Conceived of the review: PC and RC. Wrote the review: PC, DW, and RC. Analyzed literature and created tables: PC and CK. All authors edited the manuscript.

## Conflict of Interest Statement

The authors declare that the research was conducted in the absence of any commercial or financial relationships that could be construed as a potential conflict of interest.
